# Diagnostic Accuracy of ^18^F-FDG PET - CT Imaging in determining the bone marrow involvement (BMI) in pediatric Hodgkin’s Lymphoma (HL)

**DOI:** 10.12669/pjms.39.6.7721

**Published:** 2023

**Authors:** Sana Naveed, Hira Faheem, Kashif Shazlee, Muhammad Shamvil Ashraf

**Affiliations:** 1 Sana Naveed, FCPS Department of Pediatric Hematology/Oncology, Indus Hospital and Health Network, Karachi, Pakistan; 2 Hira Faheem, FCPS Department of Pediatric Hematology/Oncology, Indus Hospital and Health Network, Karachi, Pakistan; 3 Kashif Shazlee, FCPS Department of Pediatric Hematology/Oncology, Indus Hospital and Health Network, Karachi, Pakistan; 4 Muhammad Shamvil Ashraf, MRCP Department of Pediatric Hematology/Oncology, Indus Hospital and Health Network, Karachi, Pakistan

**Keywords:** PET/CT, Bone marrow biopsy, Hodgkin lymphoma, Bone marrow infiltration

## Abstract

**Objective:**

To determine diagnostic accuracy of ^18^F-FDG PET - CT imaging in determining Bone marrow involvement in pediatric HL by taking bone marrow biopsy as standard.

**Method:**

This descriptive cross-sectional study was conducted in the **Department of Pediatric Hematology/Oncology, Indus Hospital and Health Network, Karachi** from July 2021 to December 2022. Treatment naïve histologically proven pediatric HL patients of both gender and aged between two to 16 years with both ^18^F-FDG PET - CT and bone marrow biopsy imaging were included. Basic demographics such as age, gender, height, weight, as well as classification and staging of HL was obtained. Results were assessed by expert reviewers who were blinded to clinical outcome. Sensitivity, specificity, positive and negative predictive value, and diagnostic precision were assessed. The data was analyzed via SPSS 26.0.

**Results:**

Total 131 participants were included with a male predominance i.e. 104 (79.6%). The mean (±SD) age was 8.7 ± 3.4 years. The present study reported PET/CT to have a sensitivity, specificity diagnostic accuracy, PPV and NPV of 94.1%, 92%, 92%, 64% and 99% respectively.

**Conclusion:**

Our findings support the idea that BMB should not be routinely conducted in all patients but rather can be reserved exclusively for patients with dubious 18F-FDG bone marrow findings, as this test has strong diagnostic potential for evaluating BMI involvement in HL.

## INTRODUCTION

Lymphomas is considered as the most frequent cancer worldwide and constitutes 10%-15% of juvenile cancers.^1^ Hodgkin lymphoma (HL) is the second most prevalent cancer in developing nations, whereas it is the third most prevalent cancer in affluent nations.^2^ In Pakistan, HL makes up 4.9% of the population.^3^ Approximately 85% of cases of nodular lymphocyte-predominant HL are histologically classified as classic HL mixed cellularity (MC) type, a less frequent subtype.^4^ In order to tailor therapy protocols as well as stage the disease, evaluation of bone marrow infiltration (BMI) is crucial.^5^

Hematogenous spread or extension from nearby soft tissues both have the potential to cause bone marrow involvement.^6^,^7^ Involvement of the bone marrow in lymphoma patients is thought to be a symptom of a more widespread illness and to be associated with a worse prognosis.^5^ Approximately, 5-14% of patients with HL had BMI, according to studies.^8^,^9^An interesting observation was the significant bone marrow involvement seen in 23 (46%) patients from Children’s Hospital Lahore (CHL) as compared to none in Royal Marsden Hospital (RMH), UK patients (p < 0.001).^2^ Based on Ann Arbor classification with Cotswolds changes,^10^ staging for lymphoma includes a BMB and CT scan.^5^ The conventional approach for identifying bone marrow involvement is considered to be BMB of dorsal iliac crest, supplemented by MRI when necessary.^11^

The most accurate way to identify lymphoma infiltration is by a BMB, but this technique is invasive and comes with a number of risks, including bleeding and pain.^12^ For the identification of BMI in HL staging, fluoro-deoxyglucose positron emission tomography combined with CT (FDG-PET/CT) is better to iliac BMB.^13^ It can thoroughly assess condition of bone marrow & has an incredibly high sensitivity for spotting lymphoma invasion.^14^ FGD-PET/CT employing fluorine-18 (18FFDG-PET/CT) has sensitivity, specificity, and accuracy of 96%-99%, 95%-100%, and 99% respectively, for staging malignant lymphomas.^15^ As a result, numerous clinical institutes have proposed that PET-CT should be the first option for identifying lymphoma bone marrow invasion rather than bone marrow biopsy.

In view of above, our research was designed to evaluate diagnostic accuracy of FFDG-PET/CT imaging in determining BMI in pediatric HL by taking it as gold standard. To the author’s knowledge, there was relatively little literature in this field. Furthermore, because the majority of previously published studies analysed retrospective series of the HL children, there may be a high risk of bias because the researcher had to rely on information gathered by third parties that was not intended for the study. As a result, the data that was made available might not be of high quality.

The current study was carried out prospectively which may give more accurate results. The results of this study would add information in existing pool of literature by providing statistical evidence about the diagnostic accuracy which would help in bringing it in practice in place of BMB.

## METHOD

This research was carried out in a span of 18 months i.e. from July 2021 to December 2022 in the Department of Pediatric Hematology/Oncology, Indus Hospital and Health Network, Karachi after the approval of study from Institutional Review Board (IRB) of the hospital (IRD_IRB_2021_05_008; Dated: June 21, 2021). The study’s non-probability consecutive sampling technique resulted in the enrollment of 131 patients overall who met the study’s eligibility requirements. The patient’s parent or legal guardian provided their informed consent. All untreated histological proven pediatric HL patients of either gender of aged between two to 16 years who had their both PET/CT & BMB were included. The study excluded patients who had unilateral iliac crest biopsy, extra cancer, or an unclassified lymphoma. The sample size was calculated by taking the following values.^16^

**Table:** 

***For Sensitivity:*** *Sensitivity = 96%* 17	***For Specificity:*** *Specificity = 95%* 17
Prevalence of BM involvement in HL = 46%^2^	Prevalence of BM involvement in HL = 46%^2^
Margin of error = 5%	Margin of error = 5%
Confidence Interval = 95%	Confidence Interval = 95%
Sample size = 131	Sample size = 95
A sample of 131 was included	

Brief history of demographic information like age, gender, height, weight, BMI, histological distribution of HL and staging was obtained and was recorded on pre-designed proforma. All participants underwent both^18^ FFDG-PET/CT & BMB. Results were assessed by expert reviewers who were not aware of clinical outcome. Sensitivity, specificity, PPV, NPV & diagnostic precision was calculated as follows:

### Sensitivity:

Sensitivity refers to ability of ^18^FFDG: PET/CT imaging to correctly recognize patients who have BMI and was calculated using formula:


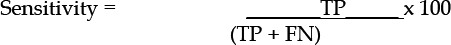


### Specificity:

Specificity relates to ability of ^18^FFDG: PET/CT imaging to correctly identify patients who do not have BMI and was calculated using formula:


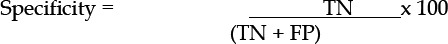


### Positive Predictive Value:

It is possibility that individuals with BMI are truly be detected by ^18^FFDG-PET/CT imaging, and was calculated using formula:


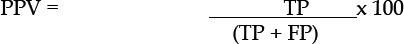


### Negative Predictive Value:

It is possibility that individuals without BMI are not detected by ^18^FFDG-PET/CT imaging and was calculated using formula:


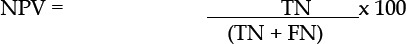


### Diagnostic Accuracy:

It was calculated by using following formula:


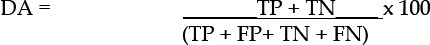


Presence of malignant nodules detected by both ^18^FFDG: PET/CT and BMB labeled the individual as true positive while their absence in both modalities marked him/her as true negative. False positive cases meant positive ^18^FFDG: PET/CT scan findings but negative BMB while negative ^18^FFDG: PET/CT scan findings but BMB findings meant false negative.

**Fig.1:**
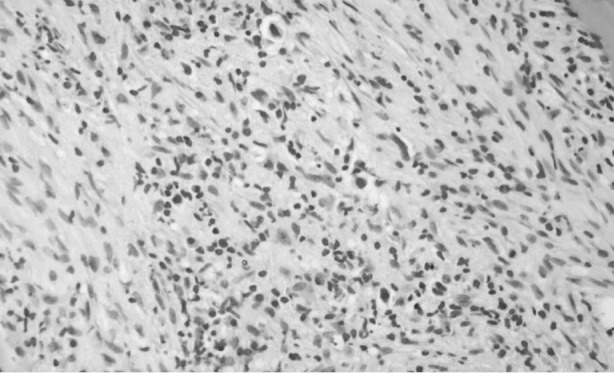
Mononuclear variant of Hodgkin cell with increased background fibrosis (H and E stained section of bone marrow trephine; 40x objective).

**Fig.2:**
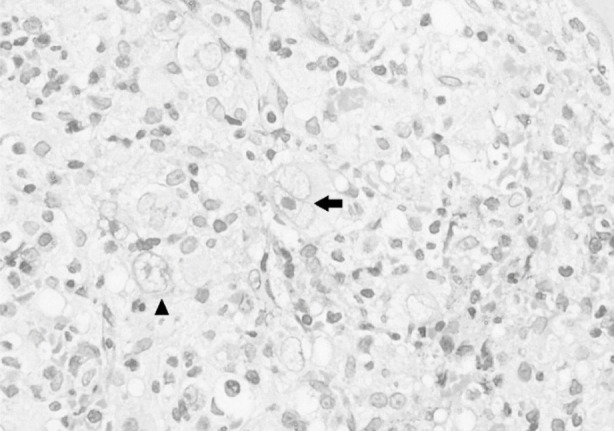
CD30 positive large Hodgkin cells. (Membranous positivity of CD30; 40x objective). Reed Sternberg cell (arrow); mononuclear cell (arrowhead).

Bone marrow involvement on ^18^F-FDG PET - CT imaging was reported as positive, if a high- uptake level of focus in bone marrow was higher than liver (or mean Standard Uptake Values of bone marrow was >2.7). Bone marrow involvement on BMB was labeled as positive if a typical Reid-Sternberg cell in a polymorphous background was observed (Fig.1& 2).

### Statistical Analysis:

The data analyzed through SPSS.26.0. Mean and Standard deviation were calculated for continuous variables such as age, height, weight and BMI while frequency and percentages were calculated for categorical variables such as gender, histological distribution of HL & staging. A 2x2 contingency table was built to evaluate the sensitivity, specificity, PPV, NPV & diagnostic precision of ^18^F-FDG PET - CT imaging in determining BMI in pediatric HL by taking BMB as gold standard.

## RESULTS

There were 131 patients in total-104 (79.6%) were male and 27 (20.4%) were female. The age range was from 2 to 16 years (8.7 ± 3.4 years). There were 34 (25.9%) were MS, 76 (58.6%) patients with NS and 22 (15.9%) patients had lymphocyte predominant HL. Most of the patients 67 (51.1%) had staging-III of HL followed by staging -IV 41 (31.2%), staging -II 22 (16.8%) and staging-I 1 (0.76%) (Table-I).

**Table-I:** Basic Demographics of patients and details Delta neutrophil index and other laboratory markers (n=131).

Variables	Frequency	Percentage
Age (mean ± SD)	8.7 ± 3.4 years	
Weight	25.57± 14.12 kg	
Height	124.34± 20.35 cm	
***Gender:***		
Male	104	79.6
Female	27	20.4
***Histological distribution of HL:***		
MS	34	25.9
NS	76	58.2
HL	22	15.9
***HL staging:***		
I	1	0.76
II	22	16.8
III	67	51.1
IV	41	31.2

Our results showed a higher sensitivity of PET/CT 94.1% (95% CI, 89.3-100%) and good specificity of 92% (95% CI, 90-100%) in detecting BMI. The diagnostic accuracy of PET/CT was 92% (95% CI, 90-99.9%) with positive predictive value (PPV) was and negative predictive value (NPV) was 64% and 99% respectively. 105/131 (80%) patients had a negative PET/CT scan for bone/bone marrow involvement along with negative BMB, which suggests that routine BMB might be unnecessary when 18F-FDG-PET/CT is negative.(Table-II)

**Table-II:** 2×2 Contingency table for determining the diagnostic accuracy of ^18^F-FDG PET – CT imaging in determining the bone marrow involvement in pediatric Hodgkin’s lymphoma (HL) by taking bone marrow biopsy as a gold standard. (n=131).

^18^F-FDG PET - CT imaging	BONE MARROW BIOPSY	

	Positive	Negative
Positive	16 (TP)	09 (FP)
Negative	1 (FN)	105 (TN)
Sensitivity	94.1%	
Specificity	92 %	
Positive Predictive Value	64%	
Negative Predictive Value	99%	
Diagnostic Accuracy	92%	

## DISCUSSION

When it comes to staging lymphoma, BMI evaluation is crucial as its existence advances the condition to Stage-IV. The results of recent research describe the effectiveness of BMB & 18F-FDG PET/CT for determining BMI in group of 131 paediatric HL cases. BMB is an invasive process that permits histologic analysis of the bone marrow taken from the posterior iliac crest. The noninvasive method 18F-FDG PET/CT, however, enables viewing of the entire bone marrow and is in contrast to this.^5^

According to our findings, PET/CT had a sensitivity, specificity, diagnostic accuracy, PPV and NPV to be 94.1%, 92%, 92%, 64%, and 99% for the identification of bone marrow involvement (BMI). According to a meta-analysis by Wu et al,^18^ which comprised of 32 trials, the sensitivity and specificity of PET/CT were 90.3% and 91.6% (95% CI, 85.9, 95.9), respectively. The detection of lymphoma’s involvement in the bone marrow by PET/CT was discovered to be a highly sensitive and specific method. However, the meta-analysis performed by Pakos et al,^19^ which involved 587 patients, the results of 18F-FDG PET/CT for diagnosing BMI did not exhibit outstanding concordance with that of BMB and displayed sensitivity and specificity of 51% (95% CI, 38-64%) and 91% (95% CI, 85-95%) respectively. This meta-analysis solely employed BMB as the gold standard, hence 18F-FDG PET/CT was not advised to take the place of the standard BMB in this meta-analysis.

In the present study, all of the patients with a positive BMB also had a positive PET/CT scan. However, there are published studies in which PET/CT identified BMI in patients that were missed by the BMB. In a study by Cheng et al.^20^ PET/CT identified BMI in two additional pediatric HL patients in a sample size of 31 cases. Agarwal et al. reported the BMI detected by FDG PET/CT in three extra patients in a study cohort of 38 pediatric HL patients that were missed by BMB giving the FDG PET/CT a sensitivity, specificity, NPV and PPV of 87.5%, 100%, 100%, and 96%, respectively.^11^

A study conducted by Purz et al. evaluated the ability to detect the BMI by F-18 FDG PET/CT and BMB in a patient population of 175 pediatric and adolescent HL patients with stage more than IIA, and reported that F-18 FDG PET/CT was able to detect BMI in 22% more cases in comparison to BMB.^21^ This led them to concluding that F-18 FDG PET may take role of BMB in normal staging procedures and was also found to be in line with other studies that advised against routinely using BMB for staging in these individuals. 22-24

In the current study, we discovered that 105/131 (80%) of patients also had negative bone/bone marrow PET/CT scans, indicating to the fact that routine BMB might not always be indicated if 18F-FDG-PET/CT is negative. Our work supports the finding of other authors previously noted that PET/CT has a strong NPV & is an effective tool for evaluating bone and bone marrow in addition to BMB.^22^

Our results are consistent with the accumulating data suggesting that BMB may eventually become unnecessary due to normal bone/bone marrow FDG uptake. Further research is necessary due to the importance of multifocal aberrant bone and bone marrow FDG uptake, which is underlined by instances where the two are inconsistent. Future research may use targeted biopsies or correlative imaging to confirm contradictory findings.

### Limitation:

The primary limitation of this study is small sample size. Future studies with large sample size are needed to confirm our observations.

## CONCLUSION

In a series of 131 paediatric patients with HL, we have presented data on 18F-FDG PET/CT and BMB performance in the detection of BMI. Our findings support the idea that BMB should not be routinely conducted in all patients but rather can be reserved exclusively for patients with dubious 18F-FDG bone marrow findings, as this test has strong diagnostic potential for evaluating BMI involvement in HL.

### Authors Contribution:

**SN:** Collected data and performed the statistical analysis, drafted the manuscript. She is also responsible for the integrity and accuracy of the study.

**HF:** Did data collection and drafted the manuscript.

**KS:** Interpreted the PET-CT and critically reviewed the manuscript for intellectual content.

**MSA:** Conceived, designed, and critically reviewed the manuscript for intellectual content.

All authors approved the final version of the manuscript.

## References

[1] Kim K, Kim SJ (2021). Diagnostic performance of F-18 FDG PET/CT in the detection of bone marrow involvement in paediatric hodgkin lymphoma:A meta-analysis. Leuk Res.

[2] Faizan M, Taj MM, Anwar S, Asghar N, Ahmad A, Lancaster D (2016). Comparison of presentation and outcome in 100 pediatric Hodgkin lymphoma patients treated at Children Hospital, Lahore, Pakistan and Royal Marsden Hospital, UK. J Coll Physicians Surg Pak.

[3] Shabbir S, Ahmed KN, Marri M, Mengal M, Jan MH, Jamali MS (2019). Epidemiological features of Lymphoma in Pakistan. Pure Appl Biol.

[4] Paulino AC, Margolin J, Dreyer Z, Teh BS, Chiang S (2012). Impact of PET-CT on involved field radiotherapy design for pediatric Hodgkin lymphoma. Pediatr Blood Cancer.

[5] McCarten KM, Nadel HR, Shulkin BL, Cho SY (2019). Imaging for diagnosis, staging and response assessment of Hodgkin lymphoma and non-Hodgkin lymphoma. Pediatr Radiol.

[6] Perry C, Lerman H, Joffe E, Sarid N, Amit O, Avivi I (2016). The Value of PET/CT in Detecting Bone Marrow Involvement in Patients With Follicular Lymphoma. Medicine (Baltimore).

[7] Skoetz N, Trelle S, Rancea M, Haverkamp H, Diehl V, Engert A (2013). Effect of initial treatment strategy on survival of patients with advanced-stage Hodgkin's lymphoma:a systematic review and network meta-analysis. Lancet Oncol.

[8] Adams H, Kwee T, De Keizer B, Fijnheer R, De Klerk J, Littooij A (2014). Systematic review and meta-analysis on the diagnostic performance of FDG-PET/CT in detecting bone marrow involvement in newly diagnosed Hodgkin lymphoma:is bone marrow biopsy still necessary?. Ann Oncol.

[9] Hines-Thomas MR, Howard SC, Hudson MM, Krasin MJ, Kaste SC, Shulkin BL (2010). Utility of bone marrow biopsy at diagnosis in pediatric Hodgkin's lymphoma. Haematologica.

[10] Hodgson D, Gospodarowicz M (2007). Clinical evaluation and staging of Hodgkin lymphoma. Hodgkin's lymphoma.

[11] Agrawal K, Mittal BR, Bansal D, Varma N, Srinivasan R, Trehan A (2013). Role of F-18 FDG PET/CT in assessing bone marrow involvement in pediatric Hodgkin's lymphoma. Ann Nucl Med.

[12] Voltin CA, Goergen H, Baues C, Fuchs M, Mettler J, Kreissl S (2018). Value of bone marrow biopsy in Hodgkin lymphoma patients staged by FDG PET:results from the German Hodgkin Study Group trials HD16, HD17, and HD18. Ann Oncol.

[13] Rogasch JM, Hundsdoerfer P, Hofheinz F, Wedel F, Schatka I, Amthauer H (2018). Pretherapeutic FDG-PET total metabolic tumor volume predicts response to induction therapy in pediatric Hodgkin's lymphoma. BMC Cancer.

[14] Weiler-Sagie M, Kagna O, Dann EJ, Ben-Barak A, Israel O (2014). Characterizing bone marrow involvement in Hodgkin's lymphoma by FDG-PET/CT. Eur J Nucl Med Mol Imaging.

[15] Zytoon AA, Mohamed HH, Mostafa BAAE, Houseni MM PET/CT and contrast-enhanced CT:making a difference in assessment and staging of patients with lymphoma. Egypt J Radiol Nucl Med.

[16] Naing L (2004). Sample size calculator for sensitivity and specificity studies. Universiti Sains Malaysia.

[17] Xiao-Xue W, Xinyue H, Lijun Z (2020). Whole body FDG-PET/CT for the assessment of bone marrow infiltration in patients with newly diagnosed lymphoma. Med Clin (Barc).

[18] Wu LM, Chen FY, Jiang XX, Gu HY, Yin Y, Xu JR (2012). 18F-FDG PET, combined FDG-PET/CT and MRI for evaluation of bone marrow infiltration in staging of lymphoma:a systematic review and meta-analysis. Eur J Radiol.

[19] Pakos EE, Fotopoulos AD, Ioannidis JP 18F-FDG PET for evaluation of bone marrow infiltration in staging of lymphoma:a meta-analysis. J Nucl Med.

[20] Cheng G, Chen W, Chamroonrat W, Torigian DA, Zhuang H, Alavi A (2011). Biopsy versus FDG PET/CT in the initial evaluation of bone marrow involvement in pediatric lymphoma patients. Eur J Nucl Med Mol Imaging.

[21] Purz S, Mauz-Korholz C, Korholz D, Hasenclever D, Krausse A, Sorge I (2011). ^18^F-Fluorodeoxyglucose positron emission tomography for detection of bone marrow involvement in children and adolescents with Hodgkin's lymphoma. J Clin Oncol.

[22] Elamir Y, Elazab M, Owis AS, Elsayed HF PET/CT and bone marrow biopsy (BMB) in evaluating bone marrow in lymphoma. Egyptian J Radiol Nucl Med.

[23] Yagci-Kupeli B, Kocyigit-Deveci E, Adamhasan F, Kupeli S (2019). The value of 18F-FDG PET/CT in detecting bone marrow involvement in childhood cancers. J Pediatr Hematol Oncol.

[24] Li Z, Li C, Chen B, Shi L, Gao F, Wang P FDG-PET/CT versus bone marrow biopsy in bone marrow involvement in newly diagnosed paediatric lymphoma:a systematic review and meta-analysis. J Orthop Surg Res.

